# A Dataset of 3D Structural and Simulated Transport Properties of Complex Porous Media

**DOI:** 10.1038/s41597-022-01664-0

**Published:** 2022-10-03

**Authors:** Javier E. Santos, Bernard Chang, Alex Gigliotti, Ying Yin, Wenhui Song, Maša Prodanović, Qinjun Kang, Nicholas Lubbers, Hari Viswanathan

**Affiliations:** 1 Los Alamos National Laboratory, Los Alamos, USA; 2 The University of Texas at Austin, Austin, USA; 3 Xii’an Jiaotong University, Xi’an, China; 4 China University of Petroleum, East China, Dongying, China

**Keywords:** Geophysics, Carbon capture and storage, Hydrology, Organic-inorganic nanostructures

## Abstract

Physical processes that occur within porous materials have wide-ranging applications including - but not limited to - carbon sequestration, battery technology, membranes, oil and gas, geothermal energy, nuclear waste disposal, water resource management. The equations that describe these physical processes have been studied extensively; however, approximating them numerically requires immense computational resources due to the complex behavior that arises from the geometrically-intricate solid boundary conditions in porous materials. Here, we introduce a new dataset of unprecedented scale and breadth, *DRP-372*: a catalog of 3D geometries, simulation results, and structural properties of samples hosted on the Digital Rocks Portal. The dataset includes 1736 flow and electrical simulation results on 217 samples, which required more than 500 core years of computation. This data can be used for many purposes, such as constructing empirical models, validating new simulation codes, and developing machine learning algorithms that closely match the extensive purely-physical simulation. This article offers a detailed description of the contents of the dataset including the data collection, simulation schemes, and data validation.

## Abstract (summary)

**Table:** 

Measurement(s)	Permeability • Conductivity • porosity • Nanoconfinement Effects • Tortuosity
Technology Type(s)	Lattice-Boltzmann Simulations • Direct 3D simulations • Image Analysis
Factor Type(s)	Lithology

## Background & Summary

Physical settings involving transport through porous media are ubiquitous in nature and industrial applications. Examples of subsurface applications include hydrocarbon recovery^1^, CO_2_ sequestration^2^, and groundwater aquifer production^3^. Further, naturally occurring examples include percolation of brine through rock salt^4^, snow and ice melt percolation through glaciers^5–8^, methane migration in marine sediments^9^, and melt migration during planetary core formation^10,11^. Some industrial applications include battery technology^12^, fibers^13^, filters, desalination^14^, and even coffee-making^15^. These examples all present challenges involving transport through porous materials. In order to forecast, design, describe, understand, or coarse-grain the relevant physics^16^, it is important to describe and quantify how different porous structures affect transport processes.

The permeability and the electrical conductivity of 3D structures are two important transport properties of great interest for the research community, particularly because analytical solutions are not available except in the most simplified, ideal geometries. The *permeability* describes how easily a fluid can travel through a particular medium. Similarly, the electrical conductivity (commonly reported as *formation factor*) quantifies how strongly the medium conducts electric current. There have been numerous efforts to develop relationships that model these properties based on the structural characteristics of the domain, but a universal relationship remains elusive^17,18^. Commonly, the porosity is used as a structural descriptor. However, plotting permeability and formation factor as a function of porosity shows significant scatter (Fig. 7). Two of the main reasons that explain this scatter are that flow and electrical properties are non-linearly affected by (1) length scale and (2) the shape of the pore-space. Geometric heterogeneities, and complex surface structures affect how open, connected, tortuous, and rough the flow paths are on a range of length scales.

Different methods can be used to estimate the transport properties of a given porous material. Laboratory measurements can provide average bulk properties, such as permeability and formation factor, of samples (typically centimeter-scale) through special core analysis measurements^19^. Three-dimensional observation of pore scale phenomena is possible using x-ray micro-tomography, though with somewhat limited ability to observe dynamic phenomena or reactive flow^20^, or by injecting tracers^21^. Another avenue for describing the flow properties of a sample is by employing published functional relationships that estimate these based on fitting parameters that account for geometrical descriptors (i.e., porosity, tortuosity, pore size distribution). While these empirical relationships are fast to evaluate, they do not provide accurate results for porous media with complex pore geometry (Fig. 7). 3D simulations using the laws of fluid mechanics provide accurate estimations of these properties but require significant computational effort.

Another promising avenue is to use machine learning (ML) models as surrogate models to estimate properties from 3D images. Current work in pore-scale ML models has shown promising results^22–27^.The high accuracy that ML models are beginning to demonstrate may allow them to be orders-of-magnitude faster surrogates for simulation flow of effective properties of pore-scale materials, or for initializing simulations into a near-converged state. Nevertheless, the pore-scale ML subspace remains vastly unexplored, in part due to the complexity of obtaining and processing data. As examples, current works are often limited to models trained with small 3D samples (<128^3^)^23^ or models trained with bigger samples (>256^3^) but with just one geometry type (spherepacks)^25^. One notable endeavor^27^ used 90,000 synthetic microstructures from 9 families of distributions downsampled to 96^3^ to perform 3D machine learning.

In order to push the state of the art for ML forward and provide comparisons between ML techniques, it would be very useful to have a large number of labeled data (the results of expensive full-physics simulations) to build models that work in the complexity of real-world pore structures. For porous media, datasets of natural examples are beginning to be assembled, but of limited scale^28,29^ and lack flow information. Large, labeled datasets are usually limited to synthetic examples^27^ and/or 2D flow. In other fields, large and diverse datasets^30,31^ have sparked revolutions in ML by providing a common focal point that allows techniques to be compared against one another. Broadly speaking, this can be attributed to two factors: Firstly, unprecedented scale and diversity of data pushes the state of the art forward. Secondly, the public availability of these datasets makes easier the comparison of different modeling techniques, so that the relative advantages and disadvantages of different approaches can be more clearly assessed. As an example in porous media, the choice of fluid solver and numerical implementation thereof is a confounding variable with unknown effects on ML model characteristics.

As such, we have set out to develop a diverse dataset of that represent challenging, complex porous media in the context of 3D simulation, empirical functional forms, and machine learning. This diversity covers dimensions such as porous media lithology, boundary conditions, geometric resolution, and physical processes simulated. In this paper, we introduce our large-scale collection of images, geometric data, and flow and electrical simulations. A large-scale collection of pore-scale data is critical for developing advanced ML algorithms that generalize to unseen samples from many real-life sources. Additionally, it (1) fosters advancement in our understanding of microscopic physics, (2) enables creation of new upscaling relationships, (3) provides a point of comparison to benchmark other simulators of physical processes, and (4) guides ML, which is still an up-and-coming field. In this paper, we report the DRP-372 dataset, which consists of 217 digital rock samples, each containing two different domain sizes (256^3^ and 480^3^). For each digital sample, there are five nanoconfinement simulations, one fluid flow simulation, ten geometric features, electrical conductivity data, topological descriptors, and png files visualizing cross-sections of the 3D data. The dataset is publicly available at^32^.

## Methods

**Fig. 1:**
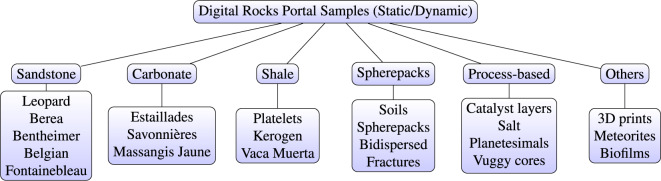
Selected samples for the dataset. The samples are divided into static, which refers to a single image, either generated synthetically or imaged with a device like an x-ray machine, whereas the dynamic ones come from imaging of experiments (i.e., fluid displacement, solid mechanics) or fluid flow simulations.

The dataset, which we refer to as DRP-372 (which stands for project number 372 from the Digital Rocks Portal), provides a comprehensive and diverse coverage of 3D samples with structural heterogeneities at different scales under different conditions. To achieve this, 125 publicly available porous media samples spanning over 50 categories/lithologies were selected from the Digital Rocks Portal (DRP)^33^ based on their quality and uniqueness. The resolution of the samples spans from 0.5 nanometers to 5 micrometers. While DRP mainly hosts subsurface rocks, it also has other materials such as catalyst layers, soils, meteorites, biofilms, and stalagmites among others. A summary of some of the included categories is shown in Fig. 1. Our dataset provides standardized image sizes, inlet/outlet orientations, and segmentation labeling of the sampled DRP data. This helps streamline ML and simulation workflows by eliminating otherwise tedious sample pre-processing steps. In the following subsections we will describe in detail how and why we selected certain geometries, the sample validation and curation, the transport simulations, and the geometric feature calculations.

### Sample selection

The workflow for imaging a sample of interest requires expert knowledge and industrial grade X-ray scanners. Several online repositories have emerged recently by providing curation and hosting services to enable the wider applicability of these technologies^29,33^. Namely, DRP provides open-access to samples from various origins (such as those in Fig. 1) imaged with a wide range of machinery. The portal provides researchers without access to imaging resources with quality peer-reviewed samples with which to work. DRP is divided into projects, which are peer-reviewed “compilations“ authored by different researchers around the globe. The samples contained in these projects were typically used in one or more journal papers. Projects often include images from different samples (e.g., rocks from different outcrops) and/or images of a specific sample under varying conditions (e.g., imaged while fluids were injected, while it was being confined with pressure, or imaged while different chemical reactions where taking place). Here, we selected 125 projects from the portal. From this selection, all the samples in each project (each project may contain any number of samples) were downloaded, standardized, and augmented. This yielded a dataset of 217 binary images - totaling more than 1.3 billion voxels - that characterize the structure each geometry. A summary description of the samples contained in our dataset is provided in Table SI.

We then performed several transport property simulations and computed geometric features on every sample. Seven 3D transport simulations were performed on each sample: electrical conductivity, five single-phase nanoconfined simulations, and one flow simulation without confinement. These simulations give rise to 3-D arrays of steady state solutions to their governing equations. The formation factor, apparent permeability, and absolute permeability (all floating point numbers) are also provided. Ten distinct 3D geometrical features were calculated, and the Minkowski functionals (5 floating points per sample) were computed. In all, the generation of this dataset took over 500 core years of computation. A detailed explanation of the image pre-processing steps (clean-up and curation), simulation procedures (processing steps, simulation methods and outputs), and geometrical property calculation, are given in the subsections below.

The entire dataset, uncompressed, is over 1 TB. It is our hope that many researchers from different scientific branches can benefit from such a large scale effort and that the scientific community can be informed of significant progress in terms of new physical insights, improved workflows, and new benchmarking algorithms.

### Geometry pre-processing

After selecting projects from DRP that maximize our dataset coverage, individual segmented images were downloaded locally. The portal allows users to upload data in a variety of formats (png, hdf5, raw, dat, tiff, numpy, among others), so we first converted each sample to a binary 3D HDF5 array^34^, where the void-space is labeled with zeros and the solid matrix with ones. Thus, each geometry in the dataset is represented by a 3D binary image. The HDF5 data format also allows for arbitrary metadata and has been shown to be efficient for digital rocks^35^; in addition, HDF5 format also allows users to build simple interfaces to access and use data as shown by Listing 1.

Multiple realizations of some of the original images are included. For example, some samples have images with sub-resolution zones, which represent areas that contained features too small to be resolved by the imaging device (these are labeled with a different number in the original dataset)^36,37^. In such cases, we created two distinct samples, one with the sub-resolution zones as pore-space and the other labeling them as solid. We also performed numerical erosions and dilations to augment the class coverage of other samples. This information is organized in Table SI.

**Fig. 2:**
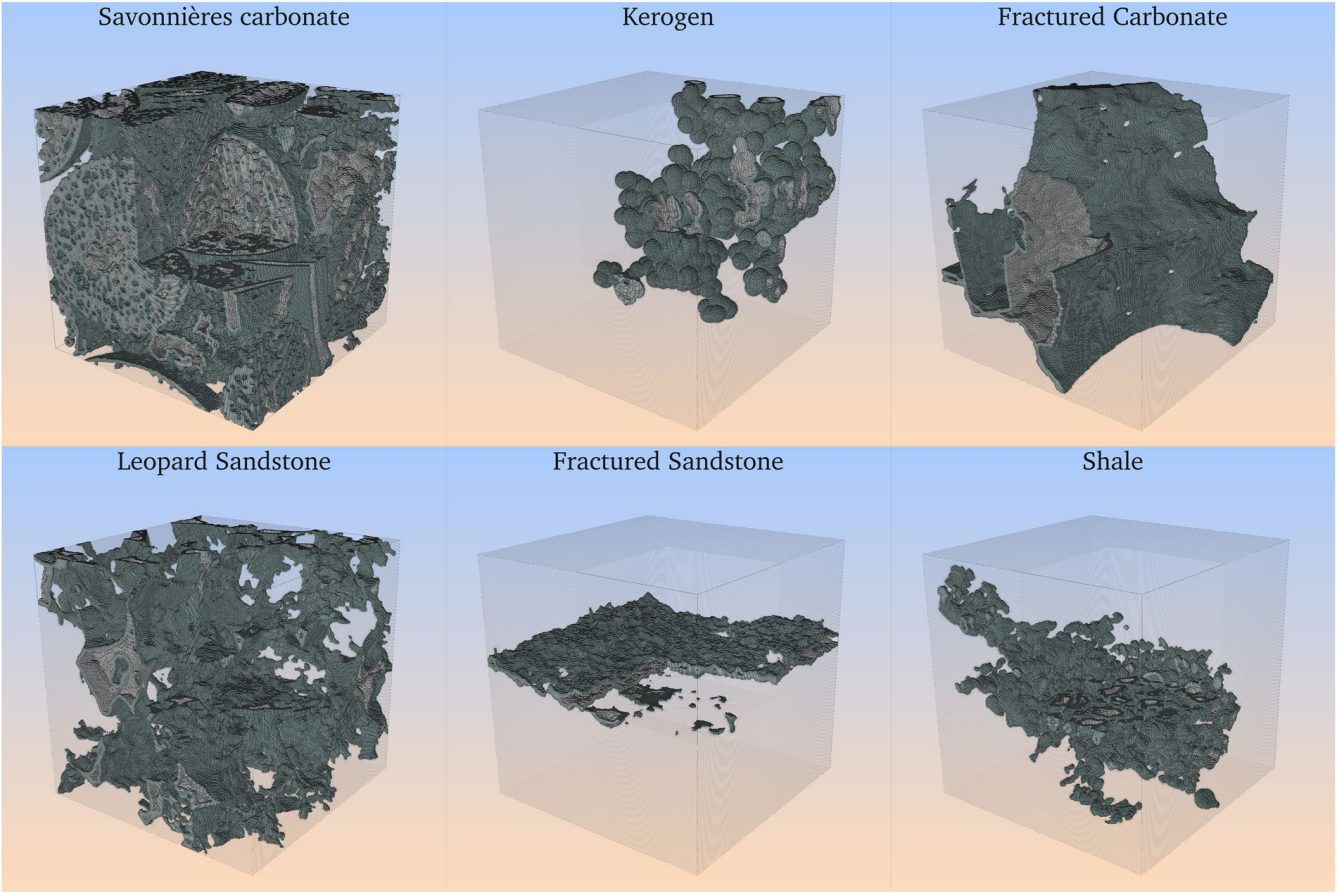
Some examples of binary geometries after the pre-processing steps. A corner of size 128^3^ was removed for visualization purposes.

We then extracted the central volume of the 3D geometry to create data items of two standardized sizes. When the source geometry is large enough, we choose a region of size 480^3^ and 256^3^; if the original image is not this large, we only sample a region of 256^3^. To ensure that the geometry was suitable for flow simulation, we performed a percolation check in the z-coordinate (third dimension) by means of a connected components algorithm: First, empty slices (labeled with zeros, according to our convention for void-space) were appended to the first and last positions along the z-axis of each sample. All connected components labeled with zeros are identified, and all but the largest (in total volume) one are erased. After the percolation check, the remaining pore-space (if any) is guaranteed to be connected in the z-direction. Those samples with no remaining pore space were then disincluded from the dataset. In the simulations, the first and last empty slices are used to set boundary conditions at the inlet and outlet, respectively. We assume that only the pore-space is available for flow and solid walls are impermeable. All the sample information is listed in Table SI and a few selected samples are show in Fig. 2.

**Fig. 3:**
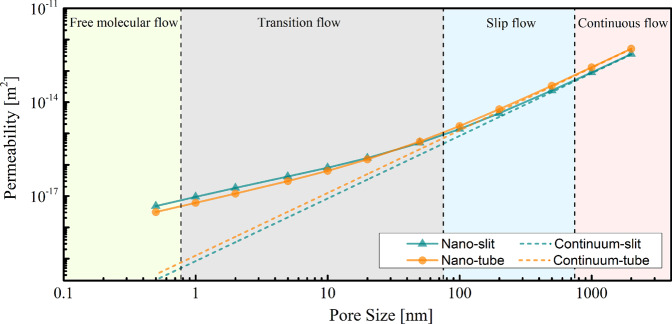
Permeability change with scale for two very simple geometries (tube and slit), demonstrating the effect of nanoconfinement in our simulation method. The x-axis denotes the pore-sizes included in our dataset. All the rest of the thermodynamic conditions are fixed. The different shaded regions correspond to free-molecular, transition, slip, and continuous (viscous) flow regimes. From the plot we can see that even in simple geometries, the change of the apparent permeability with scale is not linear and spans several orders of magnitude (due to the different physical process occurring at each scale)^80^.

All of these samples have a defined computational size of 256^3^ or 480^3^; however, it is the user’s prerogative to define the physical resolution (physical size of one voxel) of the sample. Some of the samples in the Digital Rock Portal have suggested resolutions, as they were acquired using imaging devices, which have a set resolution. The dataset also contains structures that were created synthetically (e.g., by process-based approaches^38^) that could be scaled to any resolution of interest (i.e., from nm to cm). Simulations on any geometry can also be performed at different scales to explore the impact of physics at different length-scales.

The post-processed binary images are available in the dataset. Besides their use for our simulations, these samples constitute a resource to train models to segment digital rock images. Most of the original projects have unsegmented (gray-scale) images associated with the binary images that we used here. These come from a diverse group of researchers (15 different institutions) that use various imaging machines and segmentation techniques; therefore, the dataset encompasses a diverse set of practices throughout industry and academia and would constitute a rich training resource for ML models.

### Single phase flow with and without nanoconfinement effects

Permeability is an essential measure in understanding the time scales at which fluids flow through porous media. In physical or 3D digital samples, permeability provides information about preferential flow channels and bottlenecks for fluid flow in a given domain. The permeability provides a directional, volume-averaged measure of the ease with which a fluid can flow through a sample. This quantity is impacted by the topology of the pore structures of the sample of interest and the length-scales. Permeability is calculated by computing the average fluid velocity through the pore space and comparing the velocity to Darcy’s law^39^.

In subsurface formations, the permeability is shaped by the processes that generate the rock and subsequent diagenetic alterations through geologic time. Processes such as deposition, compaction, cementation, mineral recrystallization, dissolution, and fracturing change the microscopic structure of the rock which leads to alteration of the shapes, sizes, and availability of flow paths. Subsurface formations can also host conduits and heterogeneities in a wide range of length-scales. Observations from CT and SEM imaging show features including grains, grains with microporosity, “spongy“ kerogen patches, long and skinny microfractures in materials, stacks of platelets, and so on^20^. The breadth of length scales in a porous media system can spread from features that range from pores as small as a few Angstroms across^40,41^ to fractures on the centimeter scale^42^. These scales encompass flow regimes dominated by different physical processes, from free-molecular flow to viscous-dominated flow^43^. Since the dataset contains samples across length scales, it is important that we take into account accurate fluid flow physics that encompasses the length scales of interest.

Fluid flow within systems that have nanoscale features differs from systems at larger length scales. At the nanoscale, the classical definition of fluid viscosity breaks down because the length of the travel path of a fluid molecule prior to colliding with other molecules (mean free path) is comparable to the length of the pore system. As a result, the average velocity of the fluid — and permeability of the medium — increases in comparison to larger length-scale systems. Simulation methods, such as molecular dynamics (MD), can accurately represent nanoconfinement effects. But, the computational demands for these types of simulations scale with the number of molecules in the system, which limits the calculations to very small volumes (boxes with side lengths of approximately 10 nm). These nanoconfinement effects are especially important for tight porous media like shales, where most of their storage capacity and some flow paths exist on the nanoscale.

The lattice-Boltzmann method is mesoscopic simulation approach that is able to effectively bridge scales by integrating physical insights from both the molecular- and the micro-scales. In short, fluids are simulated as swarms of particles, represented by particle distribution functions, which flow on a discrete lattice. Although, the presence of nanoscale features modifies the fluid flow behavior and poses a challenge to traditional LBM models. In response to this challenge, Landry^40^ recently proposed the local effective viscosity lattice Boltzmann method (LEV-LBM): a method that is able to capture these complicated structures and that can simulate flow regimes across multiple scales accurately. To simulate flow, LEV-LBM uses a spatially-varying mean free path, which accounts for the reduction of the local kinetic viscosity caused by the confinement effect at the nanoscale (Fig. 5).

To simulate samples at different scales, we use the local viscosity (*v*) to capture how the mean free path is affected in a confined system. This is calculated as follows:

1 $$\rho ={\mathbb{P}}{\mathbb{R}}(P,T)$$

2 $$n=\frac{\rho {N}_{Av}}{M}$$

3 $${\lambda }_{0}=\frac{1}{\sqrt{2}n\pi {d}_{m}^{2}}$$

4 $${s}_{\nu }(x)=\left(\frac{1}{2}+\sqrt{\frac{2}{\pi {c}_{s}^{2}}}{\lambda }_{0}\psi \left(x\right)\right)$$

5 $$\nu (x)={c}_{s}^{2}\left(\frac{1}{{s}_{\nu }(x)}-\frac{1}{2}\right).$$

First, the fluid density (*ρ*) is estimated using the Peng-Robinson (ℙℝ) equation of state^44^ at the desired pressure (*P*) and temperature (*T*). Using this, the number density (*n*) is calculated, where *N*_*Av*_ is the Avogadro number and *M* the molar mass. With *n*, the unbounded mean free path of the fluid (*λ*_0_) can be calculated using the hard-sphere model from kinetic theory, where *d*_*m*_ represents the diameter of the fluid molecule (i.e., 0.376 nm for methane gas). *λ*_0_ is then used to calculate the relaxation time (*s*_*v*_) for the LBM solver. *Ψ* is the 3D wall function, which calculates a normalized arithmetic mean in the 18 lattice directions at each node. *Ψ* approaches zero near the wall and increases as a function of the distance until it is equal to one (equivalent to the unbounded mean free path, hence no confinement effects). Essentially, *Ψ* represents the extent to which the flow will be affected by confinement. These parameters allow the user to simulate nano-confined and microscopic fluid flows. For example, if the wall function is set to be a 3D array of ones, this would represent a homogeneous viscosity field (not affected by confinement effects); hence this system could be representative of much larger samples (i.e., up to centimeters).

**Fig. 4:**
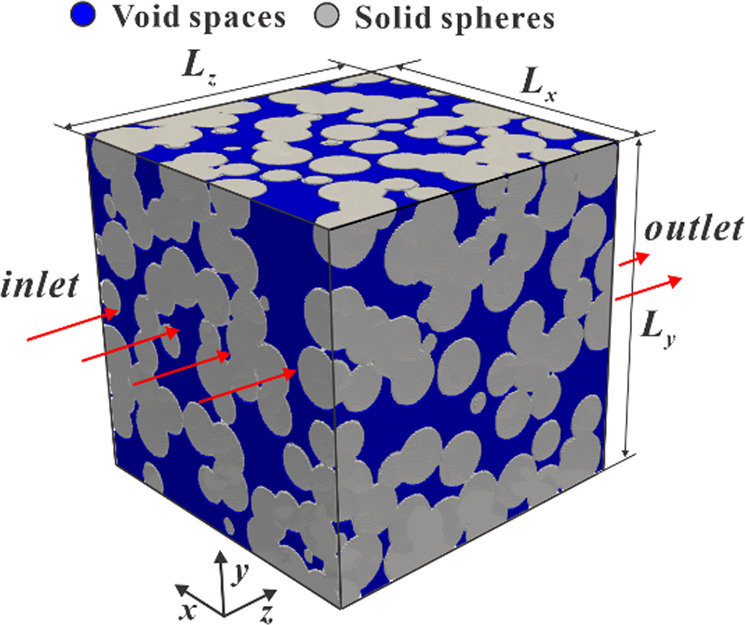
Depiction of the computation set-up for a sample of size Lx, Ly, Lz. In LB simulations, the sample is subject to a pressure gradient parallel to the z-axis, which in our case is simulated using a body force. The fluid can only travel through the void spaces (depicted in blue). In electrical simulations, the potential values at the inlet and outlet are held constant at 2 and 1, respectively; thereby subjecting the sample to a potential gradient parallel to the z-axis. Only the void space is assumed to have a non-zero conductivity value.

**Fig. 5:**
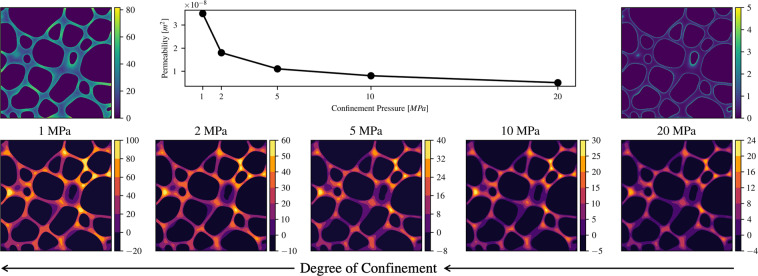
The bottom panel shows a cross-section of the nanoconfined simulation at five different pressures. The normalized velocity (with respect to the maximum value) contours are shown. It is visible that the sample at the lowest pressure (1 MPa) presents the higher degree of slip, shown as “fat“ velocity profile (fewer contour lines), while the one at 20 MPa exhibits no slip. This can also be seen in the top panels, where normalized mean free path (that could be interpreted as the deviation from the Navier-Stokes solution) is shown.

**Fig. 6:**
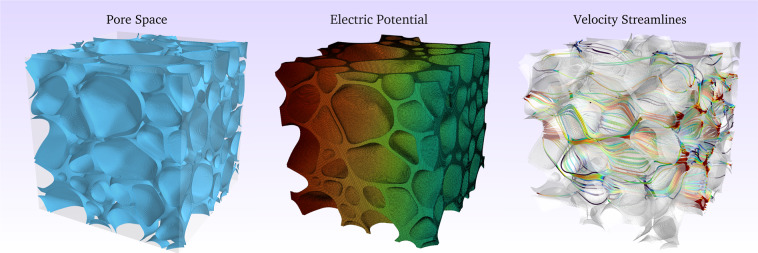
3D plots of a binary image with its corresponding electric potential simulation results and the streamline plot from the single-phase fluid flow simulation.

Both single-phase simulations with and without nanoconfinement simulations are performed using a D3Q19 lattice (three dimensions and 19 discrete velocities)^45^; to simulate a pressure drop, an external force is exerted in the z-direction, where periodic boundary conditions (which simulate a connection between the first and last slices) are applied (Fig. 4). We treat the rest of the domain faces as impermeable. In our dataset, we include the 3D pressure field and the velocity tensor (vx, vy, vz). Additionally, we provide the value of the normalized mean free path, which ranges from zero (no confinement effects) to one. Figure 5 shows a cross-section of one sample under different confinement pressures. As such, this dataset presents opportunities to study how permeability and low channels are affected by pore scale as nano-confinement sets in. These effects are non-negligible (compared to non-confined simulations) as seen on Fig. 3. A 3D schematic of the velocity streamlines of a sample can be seen in Fig. 6. These results could be representative of a wide variety of systems; batteries, catalysers, shale formations, pavements, among others.

### Electrostatic simulations

Quantification of electrical behavior in porous media has supported advancements in petroleum reservoir characterization^46,47^, CO_2_ monitoring in carbon capture and storage^48^, hydrogeology^49^, mineral exploration^50^, and battery development^51^. In these composite systems, electrical conductivity measurements aid in inferring the composition of the material and its phase distributions. For example, in petroleum systems, well-bore resistivity (reciprocal of conductivity) measurements are commonly used to estimate the amount of oil in place in the reservoir rock.

Electrical conductivity is a fundamental property of a material that quantifies how strongly it conducts electric current, where high conductivity values mean that the material readily allows current to flow. Similar to permeability, the overall electrical response of subsurface geosystems is subject to rock formation processes and subsequent diagenesis. The conductivity is primarily impacted by the topology of the conductive phase structures. Specifically, conductivity measurements capture the effects of the sinuous transport path of the connected pore space (tortuosity) and variations in the cross-sectional area of the conducting paths (constriction factor). Heterogeneities created by these processes create conductive pathways on a range of length scales similar to that of fluid flow. However, behavior at the nano- and micron-scales arguably has a more profound impact on the macroscopic (regional scale) response for electrical properties than for fluid flow. Therefore, geometric characterization of these small-scale features is crucial for inferring electrical properties on larger scales.

Many standard methods of relating the electrical conductivity to the water saturation in clay-free reservoirs are based on Archie’s empirical equations^46^. In the case where the pore space is fully saturated with conductive brine, the formation resistivity factor, *F* is useful for measuring of the influence of the pore structure on the conductivity of the sample. In Archie’s equation and subsequent formulations, *F* relates the conductivity through a rock to its porosity (*ϕ*) and a tortuosity factor (*α*) as follows:

6 $$F=\frac{{\sigma }_{w}}{{\sigma }_{o}}=\frac{a}{{\phi }^{m}},$$

where *m* is an empirical constant known as the cementation exponent and was experimentally found to be in the range of 1.8–2.0 for consolidated sandstones. *σ*_*w*_ and *σ*_*o*_ are the conductivities of the brine and fully water-saturated formation, respectively. The formation factor can be considered a normalized conductivity that provides a measure of the pore space configuration.

From Pouillet’s Law for Resistivity, the electrical conductivity of the rock is given by:

7 $${\sigma }_{{\rm{rock}}}=\frac{LI}{A\Delta V},$$

where *L* is the sample length, *I* is the total electric current, *A* is the area of a slice orthogonal to the flow of electric current, and Δ*V* is the difference in macroscopic electric potential applied to two opposite faces of the sample.

The total electric current, *I*, through the rock can be calculated as:

8 $$I=\oint \sigma \overrightarrow{\nabla }\varphi \cdot \widehat{n}dA,$$

where *φ* is the scalar electric potential field. From Ohm’s law and the continuity equation for electric flow, the resulting generalized Laplace equation for the potential field is

9 $$\overrightarrow{\nabla }\cdot (\sigma \overrightarrow{\nabla }\varphi )=0.$$

Advancements in digital rocks physics help develop a clearer picture of how complex geometries affect transport behavior^52^. Here, we use Digital Rock Suite^53^ to solve for the electric potential and current fields of each sample. In addition to the sample 3D binary images, the solver requires conductivity values to be assigned to each phase label. Every sample in this dataset contains only a grain phase and a single fluid phase. Here, we assume that solid grains are non-conductive. We, therefore, set the pore phase conductivity to 1 and the grain phase conductivity to 0. The code uses the finite difference method and the preconditioned biconjugate gradient stabilized method to discretize and solve the generalized Laplace equation for electrostatic potential. The electric potential is initialized as a linear gradient through the pore space, and the potentials at the open flow boundaries are fixed as shown in Fig. 4. The solver directs electric current in the *z* direction by only allowing electric flow in and out through the *xy* faces of the sample. Finally, the normal component of the electric current density is set to zero at conducting-non conducting interfaces (i.e. $\frac{\partial \varphi }{\partial n}=0$).

With these inputs and imposed boundary conditions, the code solves for the electric potential in each voxel. A forward difference scheme calculates the components of the electric current in the three coordinate directions. Finally, the code uses the mean current in the direction of flow to compute the bulk, macroscopic conductivity of the sample. *F* is the reciprocal of the calculated conductivity. An example of the electric potential distribution is shown in Fig. 6.

This collection of results will serve as a benchmark for simulations and as a diverse set of training data for ML models to further advance our understanding of electrical properties in porous media.

### Geometrical features

Binary images of porous materials are an important input for applications like direct simulation of physical processes. But, a 3D binary image by itself provides limited information about its overall geometric characteristics. There are many metrics that are commonly computed to characterize the structure of binary images of porous materials^54^. In this dataset, we compute ten geometrical features from each binary images described in the previous section. These features represent different aspects of the local and global topology of the original structure. These features serve as proxies for better descriptors of binary images of porous media (pore size distribution, tortuosity, local porosity), which are often used to describe sample populations. Furthermore, these features have been used as inputs for machine learning models^24,25,55–57^ to study a wide variety of relationships between structure and bulk properties of porous media. The features are grouped in the following categories:

#### Euclidean distance maps

The Euclidean distance (or *distance transform*) labels an image with the distance (in number of pixels) to the nearest solid wall. Among the features computed in this category, we include (1) the Euclidean distance of the pore-space in three coordinate directions, (2) the Euclidean distance in the XY-plane (orthogonal to the flow direction), and (3) the signed distance with positive labels inside the pore and negative labels inside the solid. Examples of each are shown in Fig. 8. These distance maps are commonly used as inputs for 3D convolutional neural network models^24,25^.

**Fig. 7:**
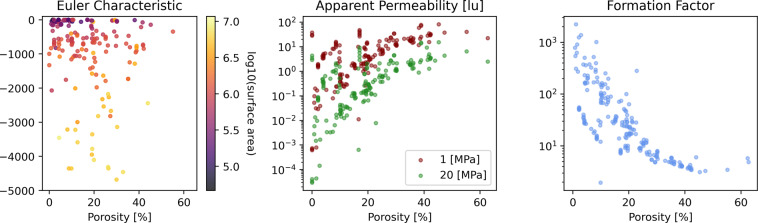
Effective properties of our dataset vs porosity.

**Fig. 8:**
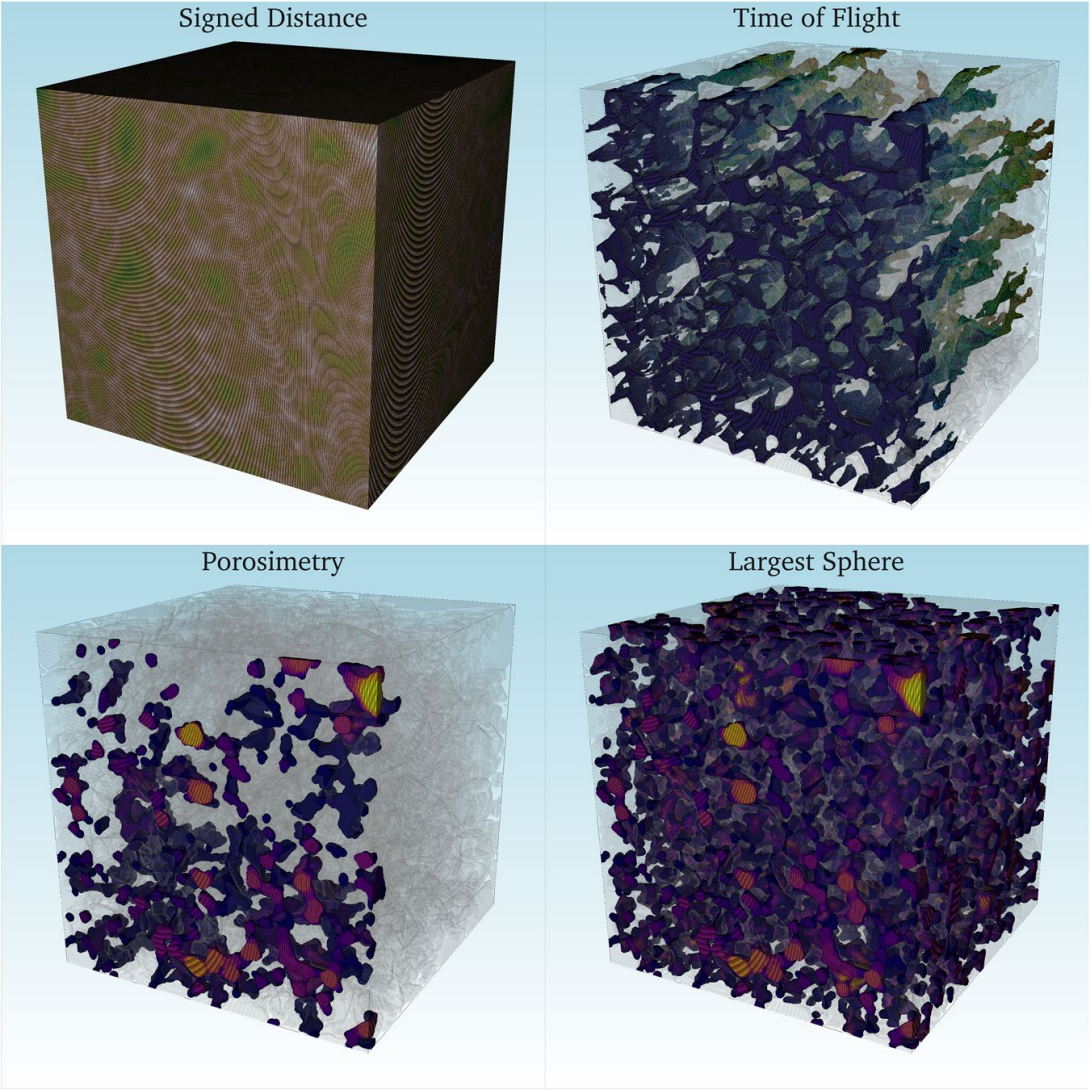
Examples of four of the computed geometrical features in our dataset, shown on a sandstone geometry. In the **signed distance**, the light brown zones correspond to the places that are far away from solid boundaries. Conversely, the darker green neighborhoods show the grain centers. The **time of flight**, from left to right is shown. We applied a threshold to the map that only shows preferential channels for flow. These correspond to the least tortuous paths that connect the boundaries. The bottom row show the features computed with inscribed spheres. In both maps we applied a threshold that goes from spheres with radii of fifteen to six (for visualization purposes). In the **porosimetry** experiment, we inscribed spheres of different sizes starting from the left boundary. Nevertheless, spheres with a radius of 6 were unable to travel through the entire domain (due to the small pore-throats), while in the **largest sphere** map those radii are enough to populate the majority of the pore-space (since this feature is not constraint and can place spheres everywhere).

#### Chord maps

One-dimensional chords can be inscribed in regions of a sample to describe its structure. In our dataset, we inscribed chords inside the pore-space in the X- and Y-directions (both orthogonal to the flow direction). This provides a map with the largest 1D segment that can be inserted at every point in a specific direction. The histogram of these provide a useful way to characterize the channel lengths inside a sample^58^.

#### Porosity

The porosity of a sample refers to the ratio of void space to the overall sample size (solid + void). The porosity of a sample is an established, oft-used structural descriptor of the void space. However, summarizing an entire heterogeneous structure with one averaged, floating point number is an oversimplification in most cases. Nevertheless, because the local porosity is one of the main factors influencing flow, we included the porosity of each slice in the z-direction. This feature is a 3D map that describes the percentage of the void volume of each slice available for flow.

#### Time of flight maps

We used the fast marching algorithm^59^ to compute the shortest distance of all the points of the domain to a plane source (In this case, both of the the XY-planes at the first and last slice, individually). This method solves the boundary value problem of the Eikonal equation. The output provides a 3D map which (1) explains how tortuous a path is (or how much a path deviates from a straight line) in the z-direction, (2) conversely also highlights the easiest paths (or *highways*) for flow, and (3) describes how connected the domain is overall. The time of flight from the left to the right boundary can be seen in Fig. 8.

#### Inscribed spheres

Spheres are commonly used in porous media applications due to their well defined geometrical properties and accurate approximations that they can provide for different phenomena^60,61^. We provide two related features. The first feature is a map with the largest sphere that can be inscribed anywhere in the void space. This map provides a 3D representation of the overall pore size distribution of the sample (tightest throats and largest pores), which tend to be of first order influence for flow (shown in Fig. 8). The second feature is a porosimetry experiment. This map is a simplified representation of a non-wetting fluid injection in the direction of flow. Although this map is typically used to describe two-phase flow, it could also act as a measure of geometry (pore sizes) and topology (connectivity to neighboring pore structures of similar size). The map provides information about the local pore space characteristics as well as the global boundaries. It acts as a bridge between the whole domain and its local regions (shown in Fig. 8).

All of these provide a comprehensive description of each sample^62^. It is our hope that new geometry-based correlations can arise from these images or their statistics. We also believe that a subset of these could be used as inputs to train ML models for different applications.

### Minkowski functionals

In addition to the previously mentioned geometric features, there are also topological methods available that can characterize a geometry. In the case of digital porous media, one can compute the Minkowski functionals (MFs), which come from the field of integral geometry and have more recently been used to characterize porous media^63–65^. Furthermore, several works have had success using MFs in finding different relationships between properties of porous materials and underlying physics^66,67^. The theoretical explanations and details on how each of the functionals is calculated can be found in a variety of sources^63–65,68^. In brief terms, Hadwiger’s theorem shows that four quantities are required to provide a complete description of 3D geometries: volume, surface area, integral mean curvature, and the total curvature (related to the Euler characteristic by the Gauss-Bonnet theorem)^64^. Together, these four quantities are known as the Minkowski functionals. It should be noted that the MFs do not provide a unique description of a geometry: there can be multiple geometric configurations that yield the same set of MFs^64^.

For this dataset we calculated the MFs of each geometry using the LBPM library^69^. The code uses the discrete formulations of the integral MF formulas, both of which can be found in the reviews by Armstrong *et al*.^64^ and Schröder-Turk *et al*.^65^. This data will engender further development of new relationships and insights that can enhance our understanding of the geometric complexities of porous media. Some future prospects include relating these topological measures to single- and multi-phase flow behavior, electrical response, and thermodynamic properties. An avenue for future research could be to use topological and geometric measures to provide a condensed yet complete description of a porous medium. ML techniques can also be employed to find new correlations between transport properties and MFs.

## Data Records

*DRP-372* has been made available on DRP^32^. Every sample image contained in our dataset is a cubic subset of a published and peer-reviewed dataset on DRP. The samples can be identified using the following naming convention: *OriginalDRPProjectNumber*_*SampleNumber*_*ImageDomainLength*. Individual samples are organized hierarchically into three levels: *Sample*, *Origin Data*, and *Analysis Data*.

- **Sample**: The top level, titled “*Sample*“ consists of the URL to the original, published dataset from which the DRP-372 sample was taken. For example sample 10 comes from https://www.digitalrocksportal.org/projects/10.
- **Origin Data**: The middle level, titled “*Origin Data*“, splits the sample by the dimensions of the subset taken from the original image. Each *Sample* consists of a 256^3^ image and a 480^3^ image. The sizes of the images are reflected in the naming convention as *ImageDomainLength*. In the following data descriptions, the side length of the image domain will be referred to as *n*. A brief description of the sample is included, detailing the image size, porosity, and whether or not percolation is achieved within the domain. This level also contains the binary images used for simulations and geometric characterization, as well as a screenshot of a 3D rendering of the pore space. The raw, binary images are saved as *SampleName.mat* and the pore space visualizations are labeled *Binary.png*.
- **Analysis Data**: The *Analysis Data* section includes the results from electrical and flow simulations and the calculated geometric features of the pore space. The results are organized by simulation type and the data contained in each are outlined below. For some samples, simulations did not achieve convergence because of non-percolating pore spaces or low porosities. Non-converging simulation results are omitted from this dataset.

The individual files per sample and the keys to access the 3D data structures are shown in Table 1.

**Table 1:** Individual files per sample. Each *.mat file (hdf5) contains several 3D arrays in double precision that can be indexed with the keys in this table.

File name	Key	Size	Description
Origin.mat	bin	*L*^3^	Curated binary geometry
LBM.mat	ux uy uz rho	*L*^3^ *L*^3^ *L*^3^ *L*^3^	x-component of flow velocity y-component of flow velocity z-component of flow velocity (flow direction) pressure field
LBM.csv	—		convergence, num of iterations, num of cores used, hours of run time, and permeability
Vel_Z.png	—		flow velocity magnitude in the direction of flow
Vel_Streamlines.png	—		streamlines calculated from the flow velocity
P_x_MPa.mat	MFP ux uy uz rho	*L*^3^ *L*^3^ *L*^3^ *L*^3^ *L*^3^	normalized mean free path x-component of flow velocity y-component of flow velocity z-component of flow velocity (flow direction) pressure field
P_x_MPa.csv	—		convergence, num of iterations, num of cores used, hours of run time, and apparent permeability
*elec.mat	Ix Iy Iz phi	*L*^3^ *L*^3^ *L*^3^ *L*^3^	x-component of electric current y-component of electric current z-component of electric current (flow direction) electric potential
Elec_Potential.png	—		visualizations of the electric potential magnitude
Elec_Streamlines.png	—		streamlines of the electric current
features.mat	chords_x chords_y e_domain e_full e_z MIS_3D MIS_z porosity_z tOf_L tOf_R	*L*^3^ *L*^3^ *L*^3^ *L*^3^ *L*^3^ *L*^3^ *L*^3^ *L*^3^ *L*^3^ *L*^3^	inscribed chords inside the pore space in the x-direction inscribed chords inside the pore space in the y-direction Euclidean distance of the pore space in all three coordinate directions signed distance function with positive labels inside the pore and negative labes inside the solid Euclidean distance in the XY-plane (orthogonal to the flow direction) maximum inscribed sphere maximum inscribed sphere in the direction of flow (drainage experiment) slice-wise porosity in the z-direction time of flight from the left boundary (inlet) time of flight from the right boundary (outlet)
features.png	—		cross-sections of five features: e_domain, tOf_L, MIS_3D, MIS_z chords_y
minkowski.csv	Vn An Jn Xn	1 1 1 1	volume surface area mean curvature Euler characteristic

We also provide a brief description of the associated *.csv and *.png files.

## Technical Validation

This section presents any experiments or analyses that are needed to support the technical quality of the dataset. This section may be supported by figures and tables, as needed. This is a required section; authors must present information justifying the reliability of their data.

We maximized the span of our dataset by including many geometry types from DRP as we show in Fig. 1. First, we ensure that each geometry was suitable for transport simulations. For each sample, percolation in the direction of flow was assessed and non-connected regions were removed. Also, samples with porosities under 1% were not used for the transport simulations. The simulators and solvers used to produce the data presented here have been individually and externally validated. For the single phase flow simulations, the LBM-LEV simulator^70^ implemented in^71^ was utilized. This simulator has been extensively validated in^40,70,72–75^. A very strict convergence criterion was utilized to ensure a very accurate approximation. Whenever the coefficient of variation of the velocity tensor between 1000 time-steps was lower than 10^−4^ the simulation was assumed to be converged. Additionally, the single-phase permeability simulation results are in excellent agreement with experiments and simulations from external groups. Each simulation was run using 144 cores for two days, if convergence was not reached, the simulation checkpoint was loaded and rerun using 576 cores in attempt to reach convergence. In all, over 90% of samples converged and the data has been made available in *DRP-372*. This process was repeated for each geometry six times with varying degrees of nanoconfinement, ranging from strongly confined to no confinement effects.

To solve for electrical properties we use Digital Rock Suite^53^. The original work validated the solver using a Finney packing of spheres and sample images of Fontainebleau sandstone. The effective conductivity of each sample was matched against values found in literature. The conductivity through a packing of spheres has been shown to be a function of sample porosity described by *σ* = *φ*^1.5^ ^76,77^. In the Fontainebleau sandstone sample, results were comparable to values reported by Doyen^78^ for the corresponding porosity range. The general assumption for simulation convergence is that slice-wise current flux remains constant throughout the sample. The solver evaluates the standard deviation of the slice-wise current flux to assess numerical uncertainties and convergence of a simulation run. In general, tighter samples do not converge to the same degree as samples with open pore spaces. The total effective conductivity is then found using the mean value of the slice-wise current flux curve. To calculate the Minkowski functionals, the LBPM software^69^ was used. This subsection of the library was been validated against multiphase flow experiments^56^.

## Usage Notes

The Usage Notes should contain brief instructions to assist other researchers with reuse of the data. This may include discussion of software packages that are suitable for analysing the assay data files, suggested downstream processing steps (e.g. normalization, etc.), or tips for integrating or comparing the data records with other datasets. Authors are encouraged to provide code, programs or data-processing workflows if they may help others understand or use the data. Please see our code availability policy for advice on supplying custom code alongside Data Descriptor manuscripts.

For studies involving privacy or safety controls on public access to the data, this section should describe in detail these controls, including how authors can apply to access the data, what criteria will be used to determine who may access the data, and any limitations on data use.

The following python code shows how to download and open the geometric and simulation data data for a given example:

**Listing 1.** Python code to download and open the data. The most up-to-date code can be found at^79^.

## Supplementary information

Supplementary Table 1

## Data Availability

The code used for the geometry curation and standarization, and plotting can be found at https://github.com/je-santos/Large-simulation-dataset. The fluid flow simulations were performed using^71^. For all studies using custom code in the generation or processing of datasets, a statement must be included under the heading “Code availability“, indicating whether and how the code can be accessed, including any restrictions to access. This section should also include information on the versions of any software used, if relevant, and any specific variables or parameters used to generate, test, or process the current dataset.
